# Inhibition of Mps1 kinase enhances taxanes efficacy in castration resistant prostate cancer

**DOI:** 10.1038/s41419-022-05312-8

**Published:** 2022-10-13

**Authors:** Sadia Sarwar, Viacheslav M. Morozov, Hamsa Purayil, Yehia Daaka, Alexander M. Ishov

**Affiliations:** 1 Department of Anatomy and Cell Biology, University of Florida College of Medicine, Gainesville, FL USA; 2 University of Florida Health Cancer Center, University of Florida College of Medicine, Gainesville, FL USA

**Keywords:** Biological sciences, Cancer, Cancer therapy, Mitosis

## Abstract

Androgen ablation therapy is the standard of care for newly diagnosed prostate cancer (PC) patients. PC that relapsed after hormonal therapy, referred to as castration-resistant PC (CRPC), often presents with metastasis (mCRPC) and is the major cause of disease lethality. The few available therapies for mCRPC include the Taxanes Docetaxel (DTX) and Cabazitaxel (CBZ). Alas, clinical success of Taxanes in mCRPC is limited by high intrinsic and acquired resistance. Therefore, it remains essential to develop rationally designed treatments for managing therapy-resistant mCRPC disease. The major effect of Taxanes on microtubule hyper-polymerization is a prolonged mitotic block due to activation of the Spindle Assembly Checkpoint (SAC). Taxane-sensitive cells eventually inactivate SAC and exit mitosis by mitotic catastrophe, resulting in genome instability and blockade of proliferation. Resistant cells remain in mitotic block, and, upon drug decay, resume mitosis and proliferation, underlying one resistance mechanism. In our study we explored the possibility of forced mitotic exit to elevate Taxane efficacy. Inactivation of the SAC component, mitotic checkpoint kinase Mps1/TTK with a small molecule inhibitor (Msp1i), potentiated efficacy of Taxanes treatment in both 2D cell culture and 3D prostasphere settings. Mechanistically, Mps1 inhibition forced mitotic catastrophe in cells blocked in mitosis by Taxanes. Androgen receptor (AR), the main driver of PC, is often mutated or truncated in mCRPC. Remarkably, Mps1i significantly potentiated CBZ cytotoxicity regardless of AR status, in both AR-WT and in AR-truncated CRPC cells. Overall, our data demonstrate that forced mitotic exit by Mps1 inhibition potentiates Taxanes efficacy. Given that several Mps1i’s are currently in different stages of clinical trials, our results point to Mps1 as a new therapeutic target to potentiate efficacy of Taxanes in mCRPC patients.

## Introduction

Prostate cancer (PC) is the second leading cause of cancer mortality in American men [1]. PC that relapses after hormonal therapies (that prevent activation of the main driver of PC, androgen receptor (AR)), is referred to as castration-resistant PC (CRPC) [2]. CRPC is the cause of almost all PC-related deaths in the U.S [1] and is often presented with metastases (mCRPC). The few available therapies for mCRPC patients include Taxanes [3–5]. Despite high resistance rates and unwanted side effects, Taxanes remain a mainstay in the clinical landscape [6].

In 2010, the FDA approved the Taxane Cabazitaxel (CBZ, Jevtana), a semi-synthetic taxane derivative, for men with mCRPC previously treated with Docetaxel (DTX), or for patients with DTX intolerance (NCCN guidelines, www.nccn.org). Nonetheless, clinical success of CBZ to treat mCRPC is limited by overt toxicity such as peripheral neuropathy [7] as well as by high intrinsic and acquired resistance rates [8, 9]. Indeed, the median time to progression after CBZ therapy in CRPC is only 2.8 months [4]. Therefore, it is essential to understand the mechanisms of CBZ resistance in order to develop rationally designed treatments for managing the so far therapy-resistant mCRPC disease. This had motivated development of new approaches, including the combination of CBZ with other drugs. CBZ combination with AR antagonists or radium-223 has the potential to benefit mCRPC patients [10]. Drug combination approaches are currently being tested in 67 out of 126 clinical trials using CBZ (https://clinicaltrials.gov).

Taxanes induce hyper-polymerization of microtubules that can affect cellular machinery at multiple levels, suggesting several mechanisms of resistance. Yet, the major effect of Taxanes on microtubule hyper-polymerization is the activation of the Spindle Assembly Checkpoint (SAC) [11] that induces mitotic block. Taxanes activity affects mitotic checkpoint [12, 13] and leads to mitotic arrest that eventually triggers cell death [14] by multiple mechanisms [15–18]. Notably, Taxane-induced hyper-polymerization of microtubules is reversible and microtubules resume normal dynamics after drug washout in cell culture settings or decay in patients. Cells resistant to Taxanes remain in mitotic block and resume mitosis after drug decay, whereas sensitive cells can react to the extended mitotic block by activating one of two complementary mechanisms. Activation of apoptosis is one of the extensive mitotic block outcomes (as described by Dr. Dixit group [19]). Apoptosis is activated by degradation of anti-apoptotic protein MCL1 by the tumor-suppressor protein FBW7, a substrate-binding component of E3 ubiquitin ligase complex. The same group demonstrated that FBW7 is deregulated in multiple tumors and cell lines and is associated with abolished taxane-induced apoptosis. Alternatively, sensitive cells can exit mitosis by a mechanism known as “mitotic slippage”, or “mitotic catastrophe” [20, 21], an event biochemically characterized by slow and steady degradation of cyclin B by the E3 ubiquitin ligase, Anaphase Promoting Complex/Cyclosome (APC/C) [20, 22, 23]. The mechanism of APC/C activation in the presence of an active SAC is not well understood. It is well established, however, that as soon as levels of cyclin B (among other APC/C substrates) drop below a threshold, cells exit mitosis in an aberrant micronucleated (MN) G1 stage, a morphological marker of mitotic catastrophe [24, 25]. MN cells often fail the next round of cell division by undergoing apoptosis, necrosis or senescence [26–28], review [29].

We reported that mild hyperthermia (HT, 41–42 °C) triggers mitotic exit/catastrophe (morphologically visualized as MN formation and biochemically as degradation of cyclin B1) in cancer cells blocked in mitosis by Taxanes treatment [30]. HT applied at the end of taxane treatment (when cells are blocked in mitosis) significantly increased the cancer cell death, including Taxane-resistant cells [30], indicating that forced mitotic exit is highly cytotoxic in Taxane-pretreated cells. We hypothesized that HT affects components of the mitotic machinery that are necessary to maintain Taxanes-induced mitotic block. Searching for targets to potentiate mitotic exit and the cytotoxic response of Taxanes, we focused on mitotic checkpoint kinase Mps1/TTK. Through phosphorylation of key SAC proteins like Bub1 and Mad1, Mps1 activates SAC to ensure metaphase to anaphase transition only upon bi-oriented attachment of all chromosomes [31]. Due to its importance for cell viability, Mps1 has emerged as a promising target for the treatment of aggressive cancers, and several Mps1 inhibitors (Mps1i) are currently in clinical trials to treat solid tumors [32–34]. Nonetheless, the efficacy of Mps1i in combination with Taxanes presents an unexplored option for PC. Our results show that Mps1i induces mitotic exit/catastrophe in CBZ-treated mCRPC cells and that Mps1i potentiates cytotoxicity of CBZ in CRPC cells in cell culture and in 3D prostasphere models. Based on these results and considering that expression of Mps1 is associated with negative prognoses in PC, our results suggest that inhibition of Mps1 activity forces mitotic exit/catastrophe of CBZ-treated mCRPC cells thereby increasing the cytotoxicity of CBZ. Hence, Mps1 inhibition can be suggested as a CBZ adjuvant therapy for mCRPC.

## Materials and methods

### Cell lines

C4-2B, 22RV1, R1-AD1, and R1-D567 [35] cells were cultured in RPMI 1640 medium with L-glutamine (Corning #10-040-CV) supplemented with 10% fetal bovine serum (Thermo Fisher Scientific #10437-036) and penicillin/streptomycin (Corning #30-002-Cl) in a humidified incubator at 37 °C with 5% CO_2_. All cell lines were tested for mycoplasma contamination.

### Antibodies

Mps1 mouse monoclonal (Abcam, #ab11108), Cyclin B mouse monoclonal (Santa-Cruz, #sc-245), H3 phospho-Ser10 rabbit monoclonal (Cell Signaling, #53348), Actin mouse monoclonal AC-74 (Sigma, #A5316), Ki-67 rabbit monoclonal (Zymed, South San Francisco, CA), PARP (Cell Signaling, #9542), Lamina and centromere (CREST) human autoimmune antibodies [36] were used.

### Colony formation assay

Cells (5000–8000) were seeded in 12-well plates, 36–40 h later treated with CBZ (C-2581 LC labs), BAY (HY-12859 MCE), DTX (D-1000, LC Labs), or a combination of the drugs for the indicated time. After treatment, cells were cultured for 7 days, fixed for 10 min with 4% formaldehyde and stained with crystal violet (0.5%). Images were acquired with Epson photo scanner and area of colonies was calculated using ImageJ software. Experiments were repeated at least three times.

### Immunofluorescence studies

Immunofluorescence was done as described [37]. Briefly, 75 × 10^4^ cells were plated on microscope coverslip glass (Fisher Scientific) in RPMI1640/10% FBS media. After treatment, cells were fixed with 1% formaldehyde for 10 min, permeabilized with 0.5% Triton X-100, and incubated with primary antibodies for 1 h at room temperature. After two washes with PBS, cells were incubated with secondary antibodies conjugated with Alexa Fluor 488 or 594 dye (Invitrogen). DNA was stained with Hoechst 33342 (Sigma). Images were analyzed using either Leica DMI4000 B fluorescent microscope or Leica TCS SP5 confocal microscope.

### Western blotting analysis

Cells were lysed directly in Laemmli sample buffer. Protein samples were separated by 4–20% SDS-PAGE (Bio-Rad) and transferred to nitrocellulose membrane using iBlot 2 system (Invitrogen, Thermo Fisher Scientific). Membranes were blocked with 5% nonfat milk/PBS, 0.1% Tween (PBST). Primary antibodies were diluted in 5% milk/PBST and incubated overnight at 4 °C. Membranes were washed three times with PBST and incubated for 1 h at ambient temperature with appropriate IRDye secondary antibody (Li-COR Biosciences). Membranes were washed three times with PBST and visualized by Odyssey CLx Imaging System (Li-COR Biosciences).

### Prostasphere growth in Matrigel

The prostaspheres were grown in Matrigel (Corning) supplemented with RPMI 1640 complete medium in 24-well plates. The basal layer was formed by mixing Matrigel and medium at a ratio 1:1; 250 μl of mixture were added per well. Cells were suspended in complete media, mixed with Matrigel at a ratio 4:1 (C4-2B) or 10:1 (R1-AD1 and R1-D567) and 200 μl of mixture with 1000 (C4-2B) and 3000 (R1-AD1, R1-D567) cells were added onto pre-solidified base layer. The plate was placed in CO_2_ tissue culture incubator to allow the upper layer to solidify. Next, 1 ml of complete media was added to each well. Prostaspheres were treated with CBZ, BAY, CBZ + BAY or CBZ next BAY on days 2 or 8 for the indicated time. Prostaspheres were stained with Calcein AM (Invitrogen) 2 weeks post-treatment (100 μl of 3.3 mM Calcein AM per well for 20 min in the tissue culture incubator). Prostaspheres were imaged using Leica fluorescent microscope and analyzed using ImageJ software. Experiments were repeated at least three times.

### Prostaspheres processing for colony formation assay

Prostaspheres in Matrigel were re-suspended in 1 ml PBS. After 3 min of centrifugation at 300 rcf, supernatant was aspirated, and cell pellet was resuspended in 0.5 ml of the remaining supernatant. Equal volume of trypsin was added for 3 min. Cells were resuspended in 4 ml RPMI complete medium and 500 cells from control prostaspheres were plated per well of 6-well plate in triplicates. The same volume of cell suspension (as used in control) was plated for each treatment condition. 3 weeks later, the colonies were fixed, stained with crystal violet, imaged and analyzed using ImageJ. Experiments were repeated at least three times.

### Staining prostaspheres sections

Histochemical staining of prostaspheres was performed according to a procedure previously described [38] with minor modification. Briefly, HistoGel™ (Thermo Scientific Richard-Allan Scientific, MI, USA) specimen processing gel was carried out at 65–70 °C for 2 h. Biopsy cryomolds were coated with pre-warmed HistoGel. The Matrigel/cell blocks separated from 8-well chamber slides were transferred onto the HistoGel pre-coated molds using a sterile scalpel. Warm HistoGel was then added on top of the Matrigel/cell block to form a sandwich. After solidification, HistoGel-Matrigel sandwiches were separated from cryomolds and packaged in paper biopsy bags (IHCworld LLC, MD, USA) that were transferred into processing/embedding pathology cassettes, fixed for 16 h in 4% formaldehyde followed by 70% alcohol, and processed for paraffin embedding. Sections (4 µm) from paraffin block were made on glass slides using a standard rotary microtome (Leica Biosystems, Deer Park, IL). Dried slides were baked at 65 °C for 1 h, deparaffinized, and used for hematoxylin and eosin (H&E) staining. For IHC, deparaffinized sections were subjected to antigen retrieval in citrate buffer (Sigma, St. Louis, MO). The sections were blocked for 30 min with protein block (Dako, Santa Clara, CA), and primary antibodies were applied as follows: Ki-67 (1:50, rabbit monoclonal; Zymed, South San Francisco, CA), H3Ser10-Phospho (1:200, rabbit; Cell Signaling, Danvers, MA), or anti-lamin, human autoimmune antibodies [36]. Fluorescent secondary antibodies were: Anti-human Alexa 594 (1:2000; Invitrogen), and anti-rabbit Alexa 498 (1:2000; Thermofisher, Waltham, MA). Cell nuclei were counterstained with 4–6-Diamidino-2-phenylindole (DAPI) (Thermofisher). Fluorescent images were obtained using Leica DMI4000 B fluorescent microscope.

### Synergism quantification

Dose-effect response for C4-2B cells was determined as described above in colony formation assay section. Drug combination effect assessment was done based on the combination index (CI) method of Chou and Talalay [39]. The graphic for dose-effect curves, combination index (CI) and the table content were generated by the CompuSyn software [40]. CI = 1, <1 and >1 indicates, respectively, additive, synergistic, and antagonistic effect.

### Senescence analysis

Cells (5 × 10^4^) were seeded in six-well plates, treated 48 h later with CBZ, BAY, and a combination of the drugs for the indicated time. After treatment, cells were grown for 7 days, fixed for 10 min with 4% formaldehyde and stained with SA-ß-gal staining solution (0.2 M citric acid/Na phosphate buffer (pH 6.0), X-gal 20 mg/ml, MgCl_2_ 1 M, NaCl 5 M, potassium ferrocyanide 30 mM, and potassium ferricyanide 30 mM) for 24 h at 30 °C and Hoechst 33342 (Sigma) for DNA staining. Images were acquired by Leica fluorescent microscope and at least 600 cells in at least five fields were analyzed with GraphPad Prism 9.2.0 as described in statistical methods section.

### Statistical methods

Statistical analysis was performed with GraphPad Prism 9.2.0 (GraphPad Software, Inc., San Diego, CA). Homogeneity of variance was estimated using Brown-Forsythe test. The comparison of means between different groups was performed by one-way ANOVA with Sidak multiple comparison test correction.

## Results

### Mps1i potentiates efficacy of CBZ treatment in CRPC cell culture

One of the few remaining treatment options of mCRPC is Taxane-based therapy that is, however, hampered by the development of resistance. Thus, it is important to rationally design combination therapy to overcome this resistance. During normal mitosis, SAC activates the mitotic kinase Mps1/TTK [41], which phosphorylates components of the mitotic checkpoint complex (MCC), Bub1 and Mad1 [31], thereby activating MCC-dependent block of APC/C. Mps1 inactivation abrogates MCC-dependent inhibition of APC/C and forces cells out of mitosis even when SAC is not satisfied, akin to the Taxane-induced mitotic block. Mps1 inhibition can force mitotic exit and, therefore, it may be used as a drug target to treat mCRPC patients. Mps1 is overexpressed in PC and its downregulation impaired growth of PC cells [42]. These results suggest that Mps1 is a promising anti-cancer target [43]. Several Mps1 inhibitors (Mps1i) have been developed and tested in vitro, in vivo and in Phase I clinical trials for breast cancer [44, 45]. Moreover, inhibition of Mps1 increases cytotoxicity of the Taxane Paclitaxel in breast cancer cells [32]. Therefore, we decided to test effect of Mps1 inhibition on Taxanes cytotoxicity in CRPC cells.

**Fig. 1:**
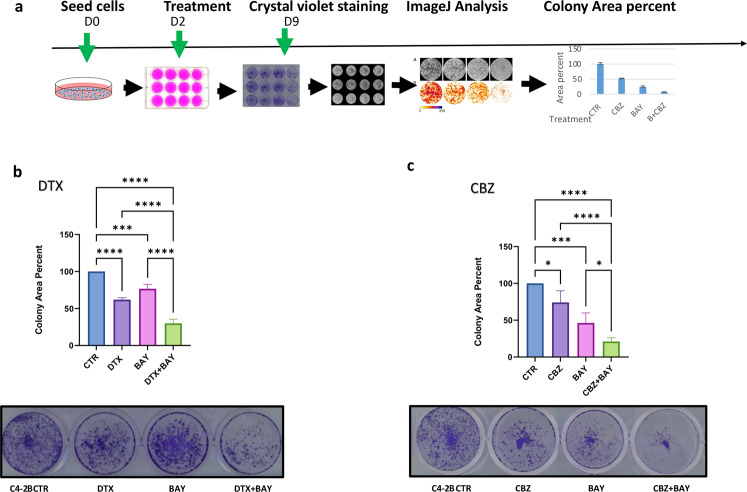
Mps1i BAY potentiates efficacy of Taxanes treatment. **a** Schematics of experiments. **b**, **c** 10^5^ C4-2B CRPC cells were plated in 12-well plate and treated 2 days later with **b** Docetaxel (DTX 0.1 nM, Mps1i BAY 0.25 nM), **c** Cabazitaxel (CBZ 0.05 nM, Mps1i BAY 2 nM) or combination for 24 h in triplicates. Colonies were stained with crystal violet 7 days later. ImageJ was used to calculate the area of the colonies (top); representative images (bottom). Experiments were repeated at least three times in triplicates; representative results are shown. In **b** and **c**, columns: mean; error bars: standard deviation. *p < 0.1; **p < 0.01; ***p < 0.001; ****p < 0.0001.

Following failure to respond to anti-androgen therapies, CRPC patients are commonly treated with DTX while CBZ is used to treat patients with intolerance or resistance to DTX. Therefore, we compared the effect of single and combination treatment of DTX and the Mps1i BAY-1217389 [32] (BAY) on C4-2B CRPC cells (Fig. 1a). We observed that combined drugs were more effective in comparison to DTX or BAY alone (Fig. 1b). We also tested the efficacy of combined treatment using CBZ. The combination of CBZ + BAY was more effective at reducing the number of C4-2B colonies compared to treatment with either CBZ or BAY alone. (Fig. 1c). We conclude that Mps1i potentiates the cytotoxic effects of both DTX and CBZ. Drug combination effect assessment was done based on the combination index (CI) method of Chou and Talalay [39]. Using the CompuSyn software [40], we observed synergistic effect of CBZ and BAY treatment (Fig. S1).

### Mps1i potentiates efficacy of CBZ treatment in CRPC cells in 3D prostasphere model

We used three-dimensional (3D) prostasphere model to confirm effect of CBZ and BAY on C4-2B growth rate. We stained sphere sections with hematoxylin and eosin (H&E) and carried out immunofluorescent analysis with proliferation marker Ki-67, mitotic marker histone H3 phospho-Ser10, nuclear lamina and DNA. Proliferating and mitotic cells were detected at the prostasphere periphery and apoptotic/necrotic cells in the sphere interior (Fig. S2).

**Fig. 2:**
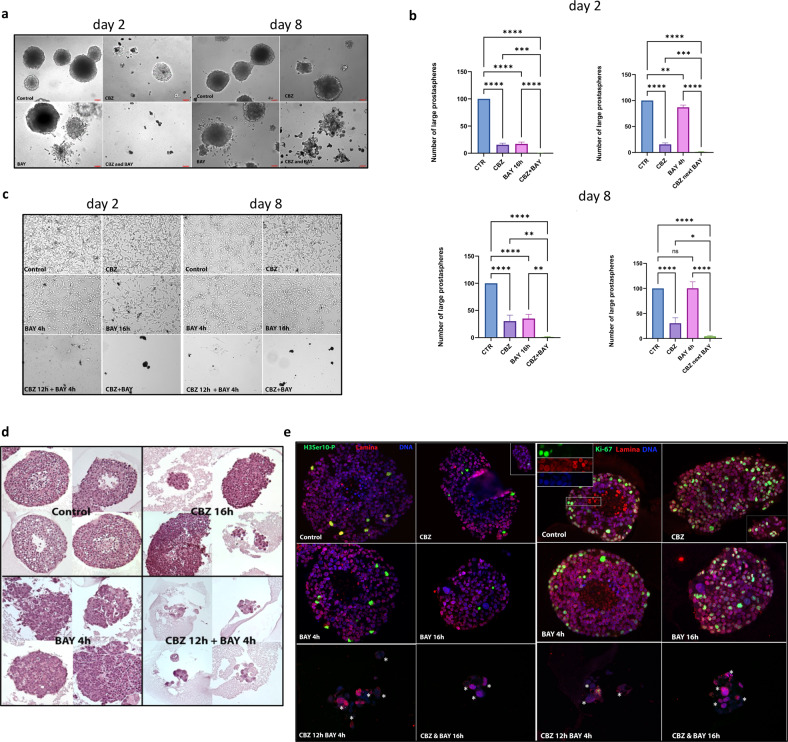
Mps1i BAY potentiates efficacy of CBZ treatment in 3D prostasphere settings. C4-2B cells were set in Matrigel and treated at day 2 or day 8 in triplicates with CBZ (1.5 nM) 16 h, BAY (32 nM) 4 h and 16 h, CBZ + BAY 16 h, CBZ 12 h next BAY 4 h. Prostaspheres were treated at day 2 (**a**, left) and day 8 (**a**, right), and documented (**a**) or counted (**b**) at day 21. In b, columns: mean; error bars: standard deviation. *p < 0.1; **p < 0.01: ***p < 0.001; ****p < 0.0001. **c**: Prostaspheres were treated at day 8; at day 21, equal volume of trypsinized cells was re-plated in six-well plates and documented 48 h later. **d**, **e** Prostaspheres were treated at day 8 as indicated and fixed 14 days later. Sections stained with: **d** H&E or **e** with H3Ser10-P (green, left panel, mitotic marker) or Ki-67 (green, right panel, proliferation marker), lamina (red), and DNA (blue); asterisk for MN cells.

Next, we tested effect of CBZ, BAY, and the combination of CBZ + BAY on prostasphere size and number at two different time points: 2 days (~4 cells/sphere) and 8 days (~100 cells/sphere). At both time points, the combined treatment obliterated the C4-2B prostaspheres formation (Fig. 2a). We also compared efficacy of combined treatment (CBZ + BAY, 16 h) with sequential (CBZ 12 h, next BAY 4 h) treatment. Rationale for sequential treatment was to evaluate the response of C4-2B cells by first blocking maximum cells in mitosis using CBZ and then forcing cells out of mitosis using BAY. Mps1 inhibition eliminated prostaspheres when applied simultaneously with CBZ or at the end of CBZ treatment (Fig. 2b). To address cell survival, we collected prostaspheres 2 weeks after treatment, and re-plated cells for colony formation. In both settings (treatment at day 2 and day 8), combined and sequential treatments were highly cytotoxic, killing almost all cells (Fig. 2c). H&E staining of prostasphere sections confirmed changed morphology and size reduction of spheres treated with CBZ + BAY (Fig. 2d). To provide mechanistic insight, we used immunofluorescence analysis of prostasphere sections stained with mitotic marker H3 phospho-Ser10, proliferation marker Ki-67, nuclear lamina and DNA to analyze cell cycle and MN formation. Both the combined and sequential treatments eliminated mitotic cells, blocked proliferation and induced mitotic catastrophe, as evidenced by MN formation (Fig. 2e, MN cells marked with asterisk). Thus, inhibition of Mps1 elevated CBZ cytotoxicity in 3D prostasphere settings.

### Characterization of combined treatment mechanism

**Fig. 3:**
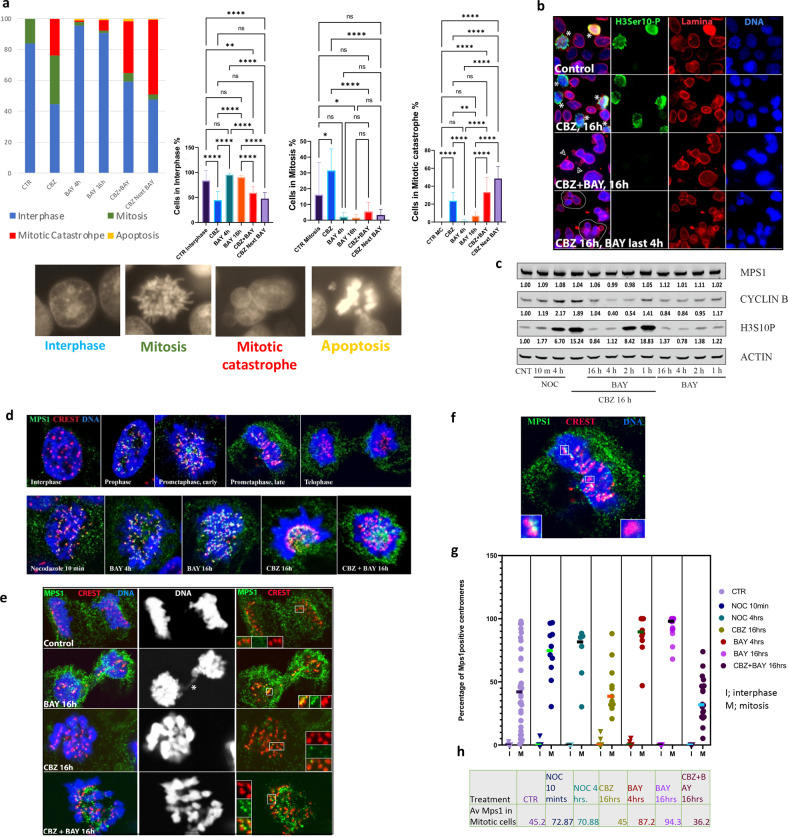
Combination of CBZ and BAY induces mitotic catastrophe in CRPC cells. **a** C4-2B cells were treated as indicated, stained with Hoechst for DNA visualization and documented. Based on DNA shape, cells were categorized as (1) interphase, (2) mitotic, (3) mitotic catastrophe/micro-nucleation, and (4) apoptotic (representative images below); at least 286 cells from 10 random fields were analyzed for each treatment condition. Left: percent of cells for each category; right: statistical analysis in individual fields, mean; error bars: standard deviation. *p < 0.1; **p < 0.01: ***p < 0.001; ****p < 0.0001. **b** C4-2B cells were treated as indicated. Immunofluorescence analysis was performed with antibodies for: H3S10P (mitotic marker, green), nuclear lamina (red), DNA was stained with Hoechst (blue). CBZ treatment accumulated cells in mitosis (asterisk), while combined and sequential treatments with BAY forced mitotic catastrophe (MN cells outlined in panel with sequential treatment). Combined treatment resulted also in micronuclei formation (arrowheads). **c** Analysis of Mps1, mitotic cyclin B1, mitotic marker H3S10-phosphorylated in C4-2B cell lysates treated as indicated. The relative levels of cyclin B, H3S10Ph and Mps1 were normalized to actin. **d**–**h** Analysis of Mps1 localization. C4-2B cells treated as indicated were stained for endogenous Mps1 (green), centromeres (red, CREST antibody), and DNA (blue, Hoechst). **d** top: Representative images of Mps1 distribution relative to centromeres throughout cell cycle. **d** bottom: Representative images of Mps1 distribution relative to centromeres after indicated treatments. **e** Analysis of Mps1 localization during mitotic exit in control and treated conditions; mitotic bridge, asterisk. **f** A representative image used for analysis of Mps1-positive (insert, left) and -negative (insert, right) centromeres. **g**, **h** Analysis of Mps1-positive centromeres. Percentage of Mps1 positive centromeres in each individual cell (categorized as interphase or mitotic) is shown in **g**, and average in **h**. In untreated cells (CTR), extensive variation of Mps1 association with centromeres indicated normal mitotic progression, while nocodazole and BAY treatment elevated association. Average number of Mps1 positive centromeres remains as in control during CBZ treatment, yet no cells were observed with association below 25%, indicating SAC inactivation and mitotic exit below this threshold. In combined CBZ + BAY treatment, association is reduced due to the forced mitotic exit.

CBZ hyper-polymerizes microtubules, thereby inducing mitotic block by SAC activation. CBZ-sensitive cells exit mitosis by a mitotic catastrophe (micronucleated (MN) morphology) whereas CBZ resistant cells remain in the mitotic block and resume proliferation after drug decay, denoting one resistance mechanism. To understand the cell fate, we analyzed nuclear morphology of cells treated with CBZ, BAY, CBZ + BAY, and CBZ then BAY. We categorized cells into interphase, mitosis, MN (lobular nuclei or several nuclei of smaller sizes), and apoptosis (highly condensed DNA). Representative images are shown in Fig. 3a, bottom. Under all treatment conditions, the number of apoptotic cells was minimal (Fig. 3a). We observed that CBZ treatment accumulated cells in mitosis, and some cells exited mitosis as MN. BAY treatment for 4 h or 16 h reduced number of mitotic cells, confirming the activity of BAY in forcing mitotic exit. MN in combined treatment was about the same as in CBZ alone, while sequential treatment elevated MN formation (Fig. 3a). To better analyze morphology of post-treated cells, we used confocal microscopy analysis of cells stained with H3 phospho-Ser10 (mitotic marker), nuclear lamina (nuclear structure marker) and DNA (Fig. 3b). As expected, we observed accumulation of mitotic cells (denoted with asterisk) after CBZ treatment. In CBZ then BAY treatment cells displayed MN morphology. In combined treatment (CBZ + BAY), in addition to MN, in a subpopulation of cells we also observed formation of 1-2 small micronuclei per cell (denoted with arrowheads), indicating loss of individual chromosomes, as expected in mitotic exit with inactivated SAC. Thus, mitotic exit by combined treatment resulted in two morphological outcomes: MN and loss of individual chromosomes, that can explain results of Fig. 3a. We conclude that CBZ and inactivation of Mps1 with BAY abrogates SAC, thus forcing mitotic catastrophe in mCRPC cells.

To support the morphological data, we used western blot to determine protein levels of Mps1, mitotic markers Cyclin B and H3 phospho-Ser10 (Fig. 3c, the full length original western blots in Fig. S3). Treatment with CBZ or Nocodazole (NOC, microtubule depolymerizing agent) induced accumulation of mitotic markers H3 phospho-Ser10 and Cyclin B1, indicating accumulation of cells in mitosis. Addition of BAY after CBZ treatment led to a reduction of both mitotic markers after 1 h of treatment and was completed at 4 h, indicating mitotic exit. Levels of both mitotic markers returned to the pre-treatment conditions at 16 h, most likely because a subpopulation of minimally damaged cells (that are expected to be partially synchronized due to the prior treatments) resumed cell cycle and entered mitosis at this time point. As expected, treatment with BAY alone did not substantially affect levels of mitotic markers. Level of Mps1 remained unchanged under all treatment conditions (Fig. 3c). Hence, we confirmed Mps1 inactivation forced mitotic exit of cells blocked in mitosis by CBZ. MN cells often fail the next round of cell division by undergoing apoptosis, necrosis or senescence [26–28]. We did not observe PARP cleavage (Fig. S4) that, together with morphological observations (Fig. 3a), indicated that apoptosis was not induced by these treatments. After mitotic exit, cells either die during the next interphase, undergo growth arrest and activate senescence, or enter cell cycle and become tetraploid with reduced survival rate [13, 17]. We performed analysis of senescence and observed that all treatments induced senescence, with highest response in the combined treatment (Fig. S5A). Most micronucleated cells (by DNA staining) are senescent (Fig. S5B). In our experimental conditions, CRPC cells activated senescence when they exited mitosis as micronucleated, thus providing a mechanism of reduced proliferation. We propose that forced exit from Taxane-induced mitotic block can overcome Taxanes resistance by senescence induction, thereby enhancing the efficacy of CRPC chemotherapy.

Mps1 phosphorylates several SAC proteins resulting in activation of mitotic checkpoint complex (MCC) that blocks APC/C. Localization at improperly attached kinetochores is required for Mps1 kinase activity. Mps1 is dissociated from kinetochores after amphitelic attachment is completed at a given chromosome, while amphitelic attachment of all kinetochores inactivates cellular Mps1 that silences the SAC/MCC, thereby inducing APC/C activation and metaphase/anaphase transition [46]. Given that kinetochore/centromere localization is required for Mps1 function, we analyzed location of endogenous Mps1 in our treatment protocols. First, we confirmed localization of Mps1 with centromeres/kinetochores throughout the cell cycle (Fig. 3d top). Mps1 was absent from centromeres in interphase cells and accumulated at majority of centromeres/kinetochores at early stages of mitosis (prophase/prometaphase). This association is gradually reduced in late prometa-metaphase and completely disappeared starting from anaphase, upon proper attachment of chromosomes and satisfaction of SAC (Fig. 3d). Mps1 accumulation at kinetochores was elevated after 10 min treatment with microtubule depolymerizing agent Nocodazole (NOC, positive control). BAY treatment induced maximum association due to the inhibition of Mps1 kinase activity. Most centromeres were Mps1-positive in CBZ-treated, and some in CBZ + BAY treated mitotic cells (Fig. 3d bottom panel).

Next, we analyzed Mps1 localization during mitotic exit (Fig. 3e). No centromeres were Mps1-positive during mitotic exit under control conditions, indicating error free progression. In BAY-treated cells, Mps1 was still associated with some centromeres, indicating improper attachment of some chromosomes and unsatisfied SAC (resulting in genomic instability, as seen by mitotic bridges (asterisk) and micronucleation). Both CBZ- and CBZ + BAY treated cells exited mitosis (indicated by de-condensation of chromosomes) without genomes partition into daughter cells. As a result of extended mitotic block during CBZ treatment, Mps1 disappeared from centromeres while in CBZ + BAY treatment, cells exit mitosis without genome partition despite Mps1 association with centromeres (Fig. 3e). Mps1-positive and -negative centromeres were counted in cells that were categorized as interphase or mitotic based on DNA shape (Fig. 3f–h). There were negligible number of centromeres positive with Mps1 in interphase cells under all conditions. In control cells, extensive variation of Mps1 association with centromeres indicated normal mitotic progression (compare with Fig. 3d, top), while NOC (10 min and 4 h) and BAY treatment elevated association. In CBZ treatment, average number of Mps1 positive centromeres remained as in control (Fig. 3h), and no cells were observed with association below 25%, indicating SAC inactivation and mitotic exit below this threshold. In combined CBZ + BAY treatment, association is reduced due to the forced mitotic exit.

### Mps1i potentiates efficacy of CBZ treatment in CRPC cell lines with different status of AR

**Fig. 4:**
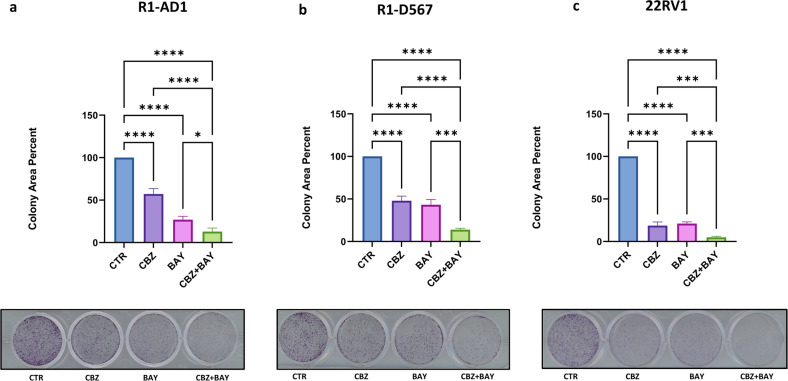
Mps1i BAY potentiates efficacy of CBZ treatment independent of AR status. Cells: R1-AD1 (AR-FL, **a**), R1-D567 (AR-DLBD, **b**) and 22Rv1 (AR-FL + AR-DLBD, **c**) were treated with CBZ (0.1 nM), BAY (3 nM R1-AD1, 1 nM R1-D567, 4 nM 22RV1) or combination for 24 h in triplicates. Colonies were stained with crystal violet 7 days later. ImageJ was used to calculate the area of the colonies (top); representative images (bottom). Experiments were repeated at least three times in triplicates; representative results are shown. In **a**–**c**, columns: mean; error bars: standard deviation. *p < 0.1; **p < 0.01; ***p < 0.001; ****p < 0.0001.

A recently identified molecular mechanism of aberrant AR activation in mCRPC is expression of AR variants (AR-Vs) [47] that lack the ligand binding domain (LBD, AR-DLBD). Clinical data and animal models confirm function of AR-Vs in CRPC [47]. Expression of AR-DLBD was identified in mCRPC [48] and facilitates treatment resistance to anti-androgens [47]. Therefore, it is important to test efficacy of combined treatment on CRPC cells which express full length AR (AR-FL) and AR-DLBD. We observed that BAY potentiates cytotoxicity of CBZ in C4-2B cells that express both AR-FL and truncated AR-DLBD (Figs. 1 and 2). Three additional cell line were included to study the role of AR in combined treatment: two isogenic cell lines with different status of AR, R1-AD1 (AR-FL) and R1-D567 (AR-DLBD) [35], and 22RV1 (expresses both AR-V7 and AR-FL [49]). First, we determined the IC50 of BAY and CBZ for each cell line. We observed that CBZ IC50 for isogenic R1-AD1 (AR-FL) and R1-D567 (AR-DLBD) were similar (0.1 nM). Next, we tested the efficacy of CBZ, BAY, and CBZ + BAY using colony formation assay. We observed that combination of CBZ + BAY was more cytotoxic than CBZ or BAY alone in all three cell lines irrespective of AR status (Fig. 4a–c).

**Fig. 5:**
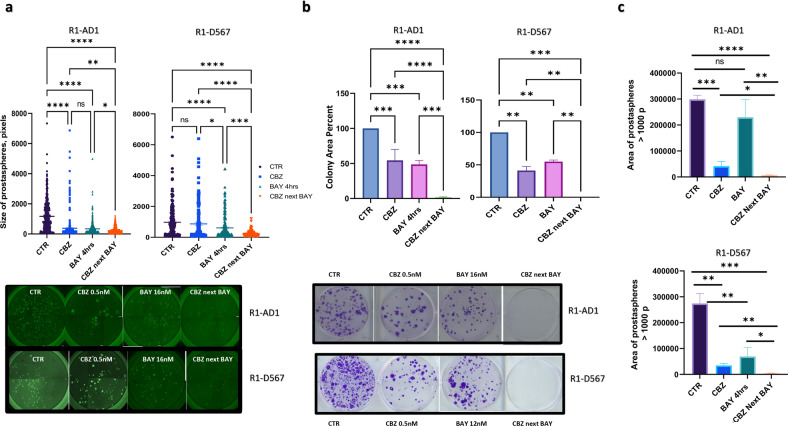
Mps1i BAY potentiates efficacy of CBZ treatment in 3D prostasphere setting independent of AR status. **a** R1-AD1 (AR-WT) or R1-D567 (AR-DLBD) prostaspheres were treated as indicated (CBZ 0.5 nM, BAY: R1-AD1 16 nM, R1-D567 12 nM) at day 8 in triplicates. At day 21, prostaspheres were stained with cell viability marker Calcein-AM, and analyzed with ImageJ and Graph PAD Prism (top; average prostaspheres size mean; bar is mean with standard error); representative images (bottom). *p < 0.1; **p < 0.01: ***p < 0.001; ****p < 0.0001. **b** Prostaspheres were treated at day 8 as indicated; at day 21, equal volume of trypsinized cells was re-plated in six-well plates in triplicates and documented four weeks later; area of the colonies was calculated with ImageJ (top); columns: mean; error bars: standard deviation. Representative images (bottom). **c** total area of prostaspheres (R1-AD1: top, R1-D567: bottom) larger than 1000 pixels (by ImageJ analysis) calculated by Graph PAD Prism, mean with standard error. *p < 0.1; **p < 0.01; ***p < 0.001; ****p < 0.0001.

We tested the efficiency of CBZ and BAY treatment in the prostasphere model in R1-AD1 (AR-FL) and R1-D567 (AR-DLBD) isogenic cells. Using combined in situ Calcein AM and propidium iodide (PI) staining, we observed that most peripheral cells were alive while most cells in the center were dead, possibly due to hypoxic conditions and poor nutrient supply (Fig. S6). These observations were similar to immunofluorescence analysis of sections of C4-2B prostaspheres (Fig. S2). We determined IC50 in prostaspheres for CBZ and BAY for both cell lines. Again, as in the 2D cell culture model, we observed a similar response to CBZ in both isogenic cell lines, further indicating that cytotoxic activity of Taxane CBZ is independent of AR status. Next, we treated R1-AD1 and R1-D567 in Matrigel with CBZ, BAY and CBZ then BAY. Prostaspheres size was analyzed two weeks later by Calcein AM staining (Fig. 5a,) using ImageJ software. Combined treatment induced significant reduction in the size of the prostaspheres in both R1-AD1 and R1-D567 cells. To address the long-term treatment effect on proliferation, prostaspheres were collected 2 weeks after treatment and cells were re-plated for colony formation (as in Fig. 2c). We observed a substantial reduction in colony numbers after single treatment with BAY or CBZ compared to control. No colonies were observed after combined drug treatment for R1-AD1 and R1-D567, confirming high cytotoxicity (Fig. 5b, top for colonies area, bottom for representative images). Our results confirmed that treatment with CBZ followed by BAY not only effectively reduced the number and size of prostaspheres, but also significantly abrogated the post-treatment cell proliferation. Comparing results of prostaspheres size and post-treatment proliferation analysis, we noticed that cells replated from wells with prostaspheres smaller than 1000 pixels (Fig. 5a) did not proliferate (Fig. 5b), suggesting that these cells, even though metabolically active (as indicated by Calcein AM staining), were unable to proliferate, potentially due to post-treatment activation of senescence. Indeed, we observed a significant reduction in the area of prostaspheres larger than 1000 pixels after CBZ next BAY treatment (Fig. 5c). Collectively, our results indicate that inhibition of Mps1 significantly potentiates CBZ cytotoxicity regardless of AR status.

## Discussion

Fast and uncontrolled proliferation is a hallmark of cancer, and therapies targeting cell cycle, including key mitotic pathways, have been at the forefront of anticancer drug discoveries [43]. Over the past decade great translational effort has been directed toward development of new drugs that block cells in mitosis by targeting the mitotic spindle assembly or function (e.g., Eg5, mitotic kinases Plk1, AurA/B, or CDKs). Alas, these drugs have shown limited efficacy and exhibited deleterious side effects in the clinic [50–52]. We hypothesized that better therapeutic effects would be expected by forcing cells to undergo mitotic exit, specifically by targeting the SAC component mitotic kinase Mps1/TTK [41]. Mps1, a key component of SAC, is essential for proper chromosome alignment and segregation during mitosis [41]. Due to its importance for cell viability, Mps1 has emerged as a promising target for the treatment of cancer. Several small molecule inhibitors of Mps1 kinase activity have been developed and are being evaluated in clinical trials [32–34]. Unlike the first generation of anti-mitotic drugs that block mitosis progression, inhibition of Mps1 forces mitotic exit and induces chromosome instability.

Searching for ways to potentiate the cytotoxic response of Taxanes, we found that inactivation of Mps1 with a small molecule inhibitor Mps1i (BAY-1217389) potentiated the efficacy of both DTX and CBZ in a 2D cell culture model (Fig. 1). We compared efficacy of sequential drug application, whereby CBZ was applied first (thus activating mitotic block) followed by BAY (presumably forcing mitotic exit) with combined CBZ + BAY treatment in a 3D prostasphere setting. We observed that BAY potentiated cytotoxic activity of CBZ under both treatment conditions (Fig. 2). Drug combination not only reduced size and number of prostaspheres, but also inhibited proliferation within prostaspheres (as indicated by the absence of mitotic cells stained with H3S10P and proliferation marker Ki-67 on spheres sections, Fig. 2e) and abrogated proliferation of cells recovered from prostaspheres (Fig. 2c), indicating the long-term cytotoxic effect of combined treatment.

Investigating mechanisms of combined treatment by microscopy and biochemical analysis, we observed that inhibition of Mps1 forces the exit of CBZ-induced mitotic block under both combined and sequential settings (Fig. 3a–c). While the total level of Mps1 protein remains unchanged in all treatment conditions (Fig. 3c), we documented a connection between kinetochore accumulation and inactivation of Mps1, thus reinforcing importance of spatiotemporal regulation of Mps1 kinase activity (Fig. 3d–h). Sequential drug treatment resulted in MN morphology, indicating forced exit from CBZ-induced mitotic block. In combined treatment, we observed formation of individual micronuclei in addition to MN (Fig. 3b) and mitotic bridges (Fig. 3e), indicating genomic instability, an expected result of mitotic progression with inactivated SAC. We speculate that the two treatment regimens potentiate CBZ cytotoxicity by activating combination of post-treatment response mechanisms, such as senescence (Fig. S5) and necrosis/necroptosis [29].

Expression of AR-Vs in mCRPC [48] facilitates treatment resistance to anti-androgens [47], and most therapy regimens in the mCRPC setting contain Taxanes. Therefore, we investigated efficacy of combined treatment on CRPC cells that express AR-FL and AR-Vs. Prior studies have demonstrated that Taxanes response may be determined by AR status via direct binding of AR [53] to microtubules via AR-hinge domain [54] that facilitates nuclear transport of AR. Other publications contradicted these results reporting that Taxanes do not act through the AR [5]. We observed no significant differences in the cytotoxicity of Taxanes DTX or CBZ between R1-AD1 (AR-FL), R1-D567 (AR-DLBD, hinge-domain positive), and 22Rv1 (AR-V7, hinge domain negative) cells. Results of combined treatment indicate that inhibition of Mps1 in both 2D cell culture and 3D prostasphere settings significantly potentiates CBZ cytotoxicity regardless of AR status.

**Fig. 6:**
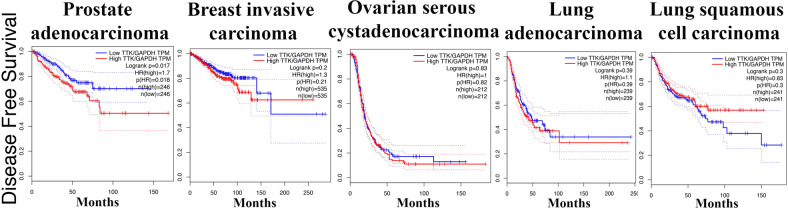
Disease free survival analysis based on Mps1 expression. Mps1 expression is normalized by GAPDH. Mps1 expression cutoff-High and -Low = 50%. Samples with expression higher or lower than 50% are considered as the high- or low-expression cohort. Logrank p test compares the survival distributions of high and low expression groups. Hazard Rate (HR) is the survival model calculated based on Cox proportional hazards model. HR > 1 indicates reduced survival of patients with high expression of Mps1. High expression of Mps1 is associated with reduced disease-free survival of prostate adenocarcinoma patients. No correlation between levels of Mps1 and patients survival observed in breast, ovarian and lung cancer patients. Data analyzed at: http://gepia.cancer-pku.cn.

Due to its importance for cell viability, Mps1 inhibitors are currently in clinical trials for breast cancer. Analyzing levels of Mps1 in solid cancer patients treated with Taxanes, we observed no correlation with disease-free survival in breast or ovarian carcinoma, and lung adenocarcinoma patients (Fig. 6, http://gepia.cancer-pku.cn). High levels of Mps1 has positive correlation with disease-free survival in lung squamous cell carcinoma patients, while it has negative correlation in prostate adenocarcinoma, further suggesting Mps1 as a target in PC and mCRPC.

**Fig. 7:**
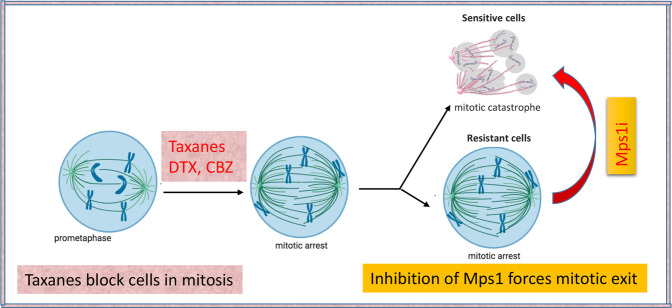
Model. Antimitotic drugs including the Taxane CBZ induce mitotic block. Sensitive cells exit block by mitotic catastrophe, while resistant cells remain in block and complete mitosis after drug decay. Mps1 inhibitors force mitotic exit inducing mitotic catastrophe, thus potentiating cytotoxicity and reversing resistance to CBZ.

In this study we aimed to characterize novel treatment combination to enhance the efficacy of Taxanes in mCRPC. Our results showed that inhibition of the SAC kinase Mps1 by a small molecule BAY together with Taxanes reduced growth rate of cells in cell culture and in prostasphere models. Mechanistically, we demonstrated that inhibition of Mps1 activity forces mitotic exit/catastrophe of CBZ-treated mCRPC cells, thereby increasing the cytotoxicity of this anticancer drug (model in Fig. 7). Considering that accumulation of Mps1 correlates with negative prognosis in prostate adenocarcinoma patients, this novel combination treatment can bring significant advancement in the clinical management of mCRPC by effectively killing CBZ resistant mCRPC tumor cells and reducing the CBZ dosage in combination with Mps1i to minimize the cytotoxicity caused by Taxanes treatment.

## Supplementary information

Supplementary material fig legends

Fig S1

Fig S2

Original Data File

Original Data File

Fig S5

Fig S6

A reproducibility checklist

## Data Availability

All data generated or analyzed during this study are included in this published article and its supplementary information files.
