# Supplementary material for: Inhibition of Mps1 kinase enhances taxanes efficacy in castration resistant prostate cancer
Source: Cell Death Dis. 2022 Oct 13;13(10):868. doi: 10.1038/s41419-022-05312-8 (PMC9561175; doi:10.1038/s41419-022-05312-8)
Supplement: Supplementary file 1 — Supplementary material fig legends [file 41419_2022_5312_MOESM1_ESM.docx]

**Fig. S1. Analysis of drug combination effect.** The graphic for dose-effect curves, combination index (CI) and the table content were generated by the CompuSyn software. CI=1, <1 and >1 indicate, respectively, additive, synergistic and antagonistic effect. Treatment of C4-2B cells with a combination of CBZ and BAY shows synergistic effect with a CI value ranging from 0.692 to 0.893.

**Fig. S2.** **Characterization of prostasphere model.** C4-2B cells were set up in Matrigel. After three weeks in culture, prostaspheres were fixed. Left image: section was stained with hematoxylin and eosin (H&E). Right image: section was stained with H3 phospho-Ser10 (bright green, mitotic marker), Ki-67 (spotted green, proliferation marker), lamina (red), and DNA (blue).

**Fig. S3.** **The full length original Western blots for Fig. 3c.**

**Fig. S4. PARP cleavage analysis.** Western blot analysis of PARP in C4-2B cell lysates treated as indicated. No PARP cleavage was observed under any treatment condition. Actin was used as a protein loading control.

**Fig. S5. Senescence test (**ß-gal staining)**.** **A:** Graph showing percentage of senescence C4-2B cells after treatment with CBZ, BAY, or a combination of these drugs. At least 600 cells in at least five randomly chosen fields were analyzed. Blue cells were considered senescent. **B:** representative images of C4-2B cells (Control and treated with CBZ+BAY) stained for β-gal as a senescence marker (upper panel) and DNA (lower panel).

**Fig. S6.** **Optimization of prostasphere model.** Prostaspheres were documented with (left) phase contrast, and (right) staining with Calcein AM (cell viability marker, green) and propidium iodide (dead cells, red). Prostaspheres had most live cells in the periphery and dead cells in the center.
