# Supplementary material for: Inhibition of Mps1 kinase enhances taxanes efficacy in castration resistant prostate cancer
Source: Cell Death Dis. 2022 Oct 13;13(10):868. doi: 10.1038/s41419-022-05312-8 (PMC9561175; doi:10.1038/s41419-022-05312-8)
Supplement: Supplementary file 2 — Fig S1 [file 41419_2022_5312_MOESM2_ESM.pdf]

Fig. S1

Dose-effect Curve

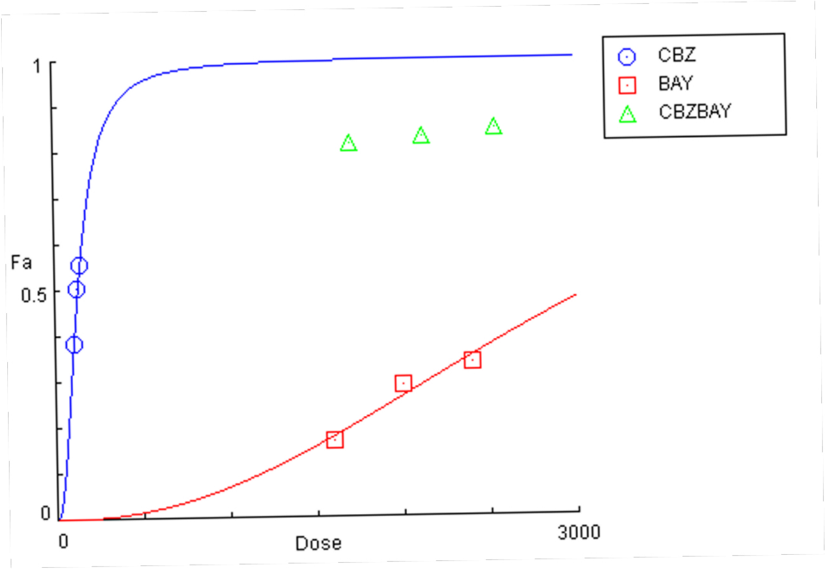

Combination Index

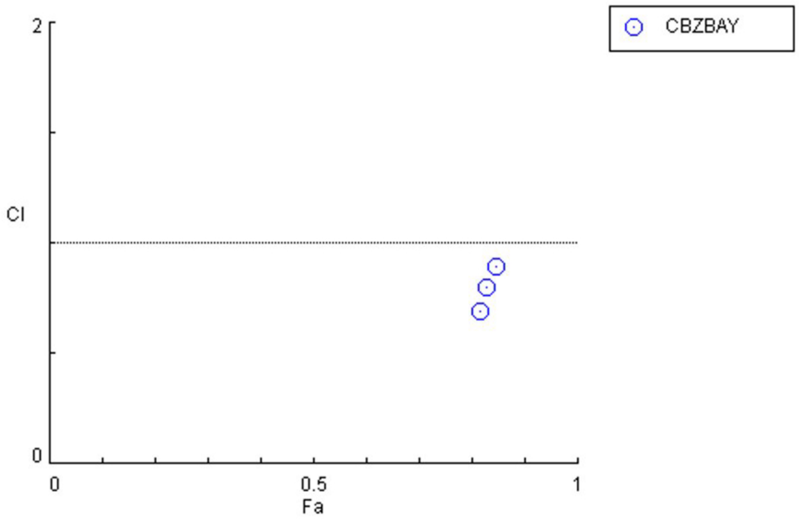

CBZ Dose pM	Effect	BAY Dose pM	Effect	CBZBAY Combination pM	Effect	Combination Index
110	**0.3843**	1600	**0.1689**	110+1600	0.8165	**0.69243**
130	**0.5057**	2000	**0.2894**	130+2000	0.8298	**0.80346**
150	**0.556**	2400	**0.3362**	150+2400	0.8463	**0.89269**
