# Supplementary figures and images for: Inhibition of Mps1 kinase enhances taxanes efficacy in castration resistant prostate cancer
Source: Cell Death Dis. 2022 Oct 13;13(10):868. doi: 10.1038/s41419-022-05312-8 (PMC9561175; doi:10.1038/s41419-022-05312-8)

Fig. S2

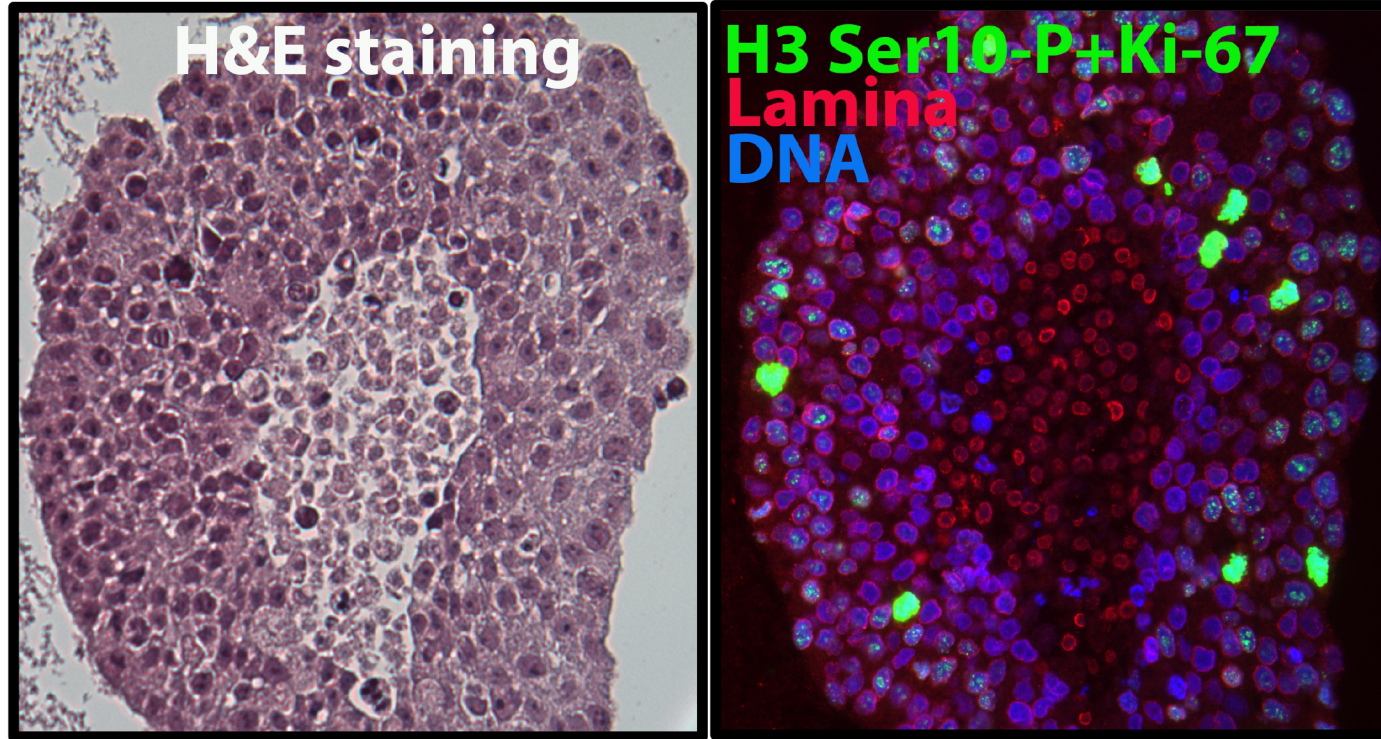

Supplement: Supplementary file 3 — Fig S2 [file 41419_2022_5312_MOESM3_ESM.pdf]

Fig S3

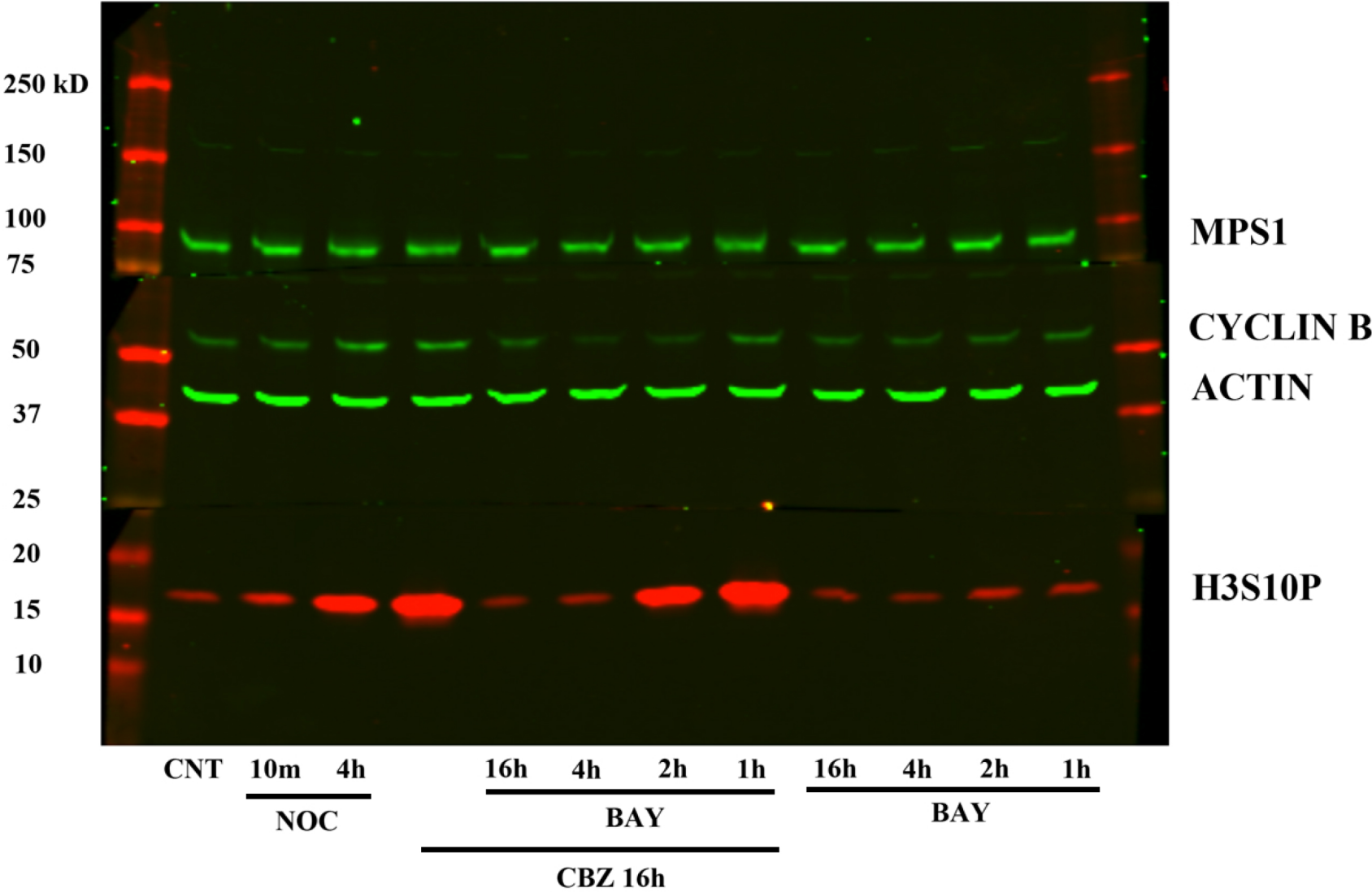

Supplement: Supplementary file 4 — Original Data File [file 41419_2022_5312_MOESM4_ESM.pdf]

**Fig. S4**

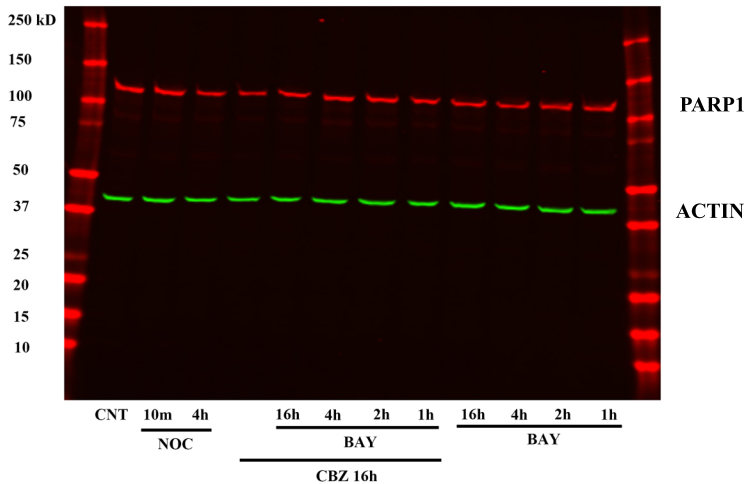

Supplement: Supplementary file 5 — Original Data File [file 41419_2022_5312_MOESM5_ESM.pdf]

Fig. S5

A

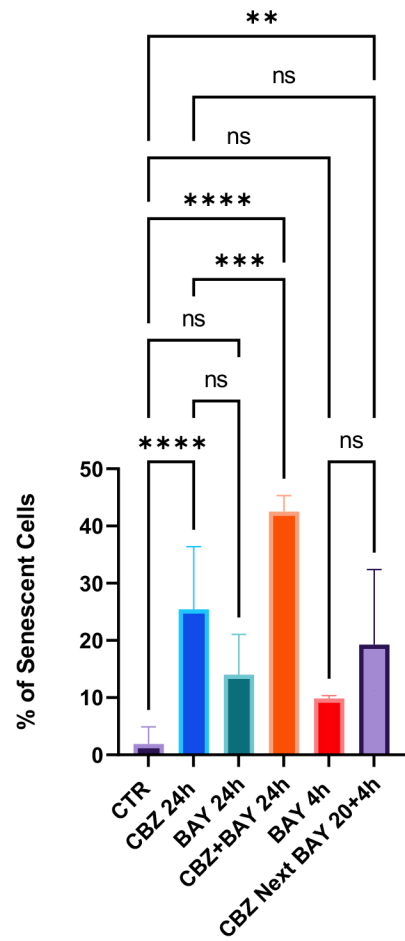

B

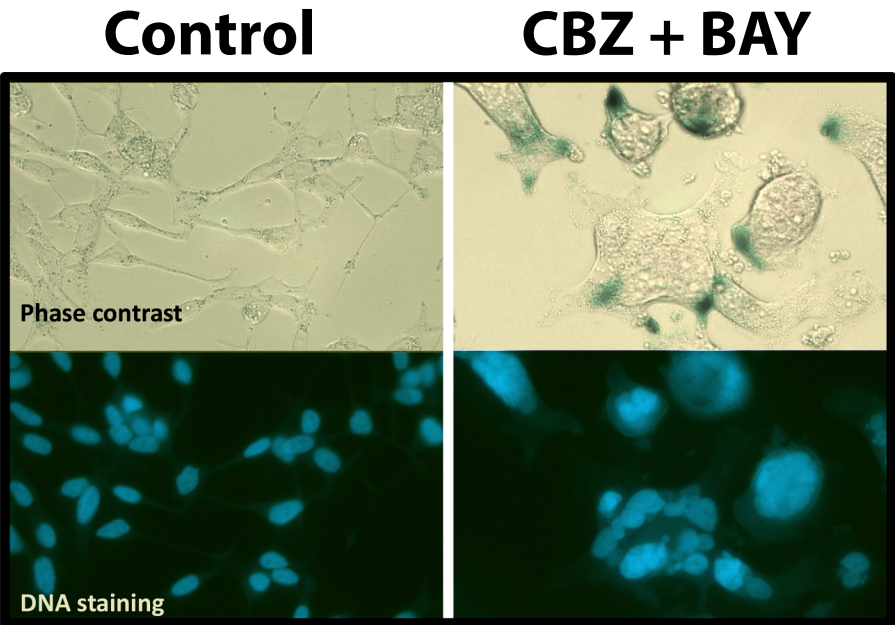

Supplement: Supplementary file 6 — Fig S5 [file 41419_2022_5312_MOESM6_ESM.pdf]

**Fig. S6**

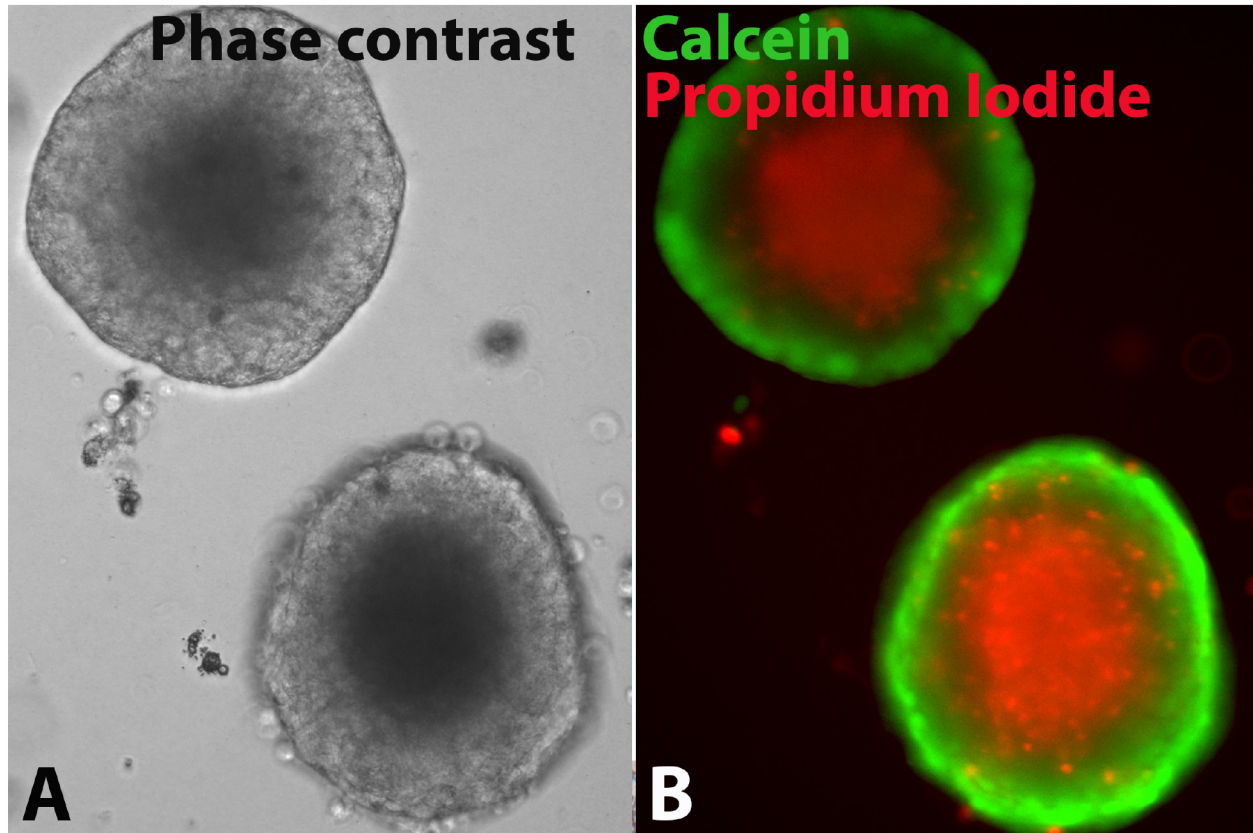

Supplement: Supplementary file 7 — Fig S6 [file 41419_2022_5312_MOESM7_ESM.pdf]
